# Centre‐level variation in behaviour and the predictors of behaviour in 5‐year‐old children with non‐syndromic unilateral cleft lip: The Cleft Care UK study. Part 5

**DOI:** 10.1111/ocr.12187

**Published:** 2017-06-29

**Authors:** A. Waylen, O. Mahmoud, A. K. Wills, D. Sell, J. R. Sandy, A. R. Ness

**Affiliations:** ^1^ Bristol Dental School University of Bristol Bristol UK; ^2^ School of Social and Community Medicine University of Bristol Bristol UK; ^3^ North Thames Regional Cleft Service Speech and Language Therapy Department and Centre for Outcomes and Experience Research in Children's Health, Illness and Disability (ORCHID) Great Ormond Street Hospital NHS Foundation Trust London UK; ^4^ National Institute for Health Research (NIHR) Biomedical Research Unit in Nutrition Diet and Lifestyle at the University Hospitals Bristol NHS Foundation Trust and the University of Bristol Bristol UK; ^5^ Department of Applied Statistics Helwan University Cairo Egypt

**Keywords:** child behaviour, cleft, psychosocial factors, SDQ

## Abstract

**Objectives:**

The aims of this study were to describe child behavioural and psychosocial outcomes associated with appearance and speech in the Cleft Care UK (CCUK) study. We also wanted to explore centre‐level variation in child outcomes and investigate individual predictors of such outcomes.

**Setting and sample population:**

Two hundred and sixty‐eight five‐year‐old children with non‐syndromic unilateral cleft lip and palate (UCLP) recruited to CCUK.

**Materials and methods:**

Parents completed the Strengths and Difficulties questionnaire (SDQ) and reported their own perceptions of the child's self‐confidence. Child facial appearance and symmetry were assessed using photographs, and intelligibility of speech was derived from audio‐visual speech recordings. Centre‐level variation in behavioural and psychosocial outcomes was examined using hierarchical models, and associations with clinical outcomes were examined using logit regression models.

**Results:**

Children with UCLP had a higher hyperactive difficulty score than the general population. For boys, the average score was 4.5 vs 4.1 (*P*=.03), and for girls, the average score was 3.8 vs 3.1 (*P*=.008). There was no evidence of centre‐level variation for behaviour or parental perceptions of the child's self‐confidence. There is no evidence of associations between self‐confidence and SDQ scores and either facial appearance or behaviour.

**Conclusions:**

Children born with UCLP have higher levels of behaviour problems than the general population.

## 1 INTRODUCTION

In the general population, most children progress through childhood and adolescence without any major behavioural problems. They may be more or less well behaved and they are likely to fall in and out of friendships but for the majority, such issues resolve over time.

While there is some evidence that children with chronic health conditions,^1^, ^2^, ^3^ craniofacial conditions generally^4^, ^5^ and those born with cleft lip and palate [CLP]^6^, ^7^, ^8^, ^9^, ^10^ are more likely to experience behavioural problems than age‐ and gender‐matched norms, a systematic review from 2005 concluded that most children with CLP do not experience major psychosocial problems.^1^ However, the likelihood of conduct problems appears to increase as children get older. There is also some evidence of differences in the likelihood and type of behavioural problems according to cleft subtype. Children born with a cleft lip and palate (rather than a cleft of either the lip or the palate) may be more likely to have a negative outlook, report negative self‐worth and to be more hostile in relationships.^1^ Similarly, those born with a cleft lip are least likely to experience behaviour or emotional difficulties, those with an isolated cleft palate are more likely to have social difficulties and those born with a cleft of both lip and palate are reported to be less competent overall with reduced social, academic and activity competencies.^1^, ^5^

It is unclear whether reported behavioural problems are a consequence of the condition of CLP or due to differences in key clinical outcomes such as facial appearance and speech.^6^ Infants born with CLP are likely to look different and they may find it difficult, not only to feed but also to communicate in infancy. These issues may be associated with difficulties in the bonding relationship between mother and infant^11^, ^12^ and the infant's developing ability to self‐regulate and control negative emotions.^13^, ^14^ Children who are unable to control their own negative emotions may have more internalizing and behavioural problems than those who can^7^ and this inability to adjust is associated with poor peer relationships and an increased risk of social teasing.^8^, ^9^ Problematic peer relationships may also arise as a result of the multiple functional and aesthetic surgeries that children born with CLP undergo to rectify dental, speech and appearance issues^15^ (cited in Brand, 2009 #4552).^16^

Despite evidence that childhood behavioural problems may be a characteristic of children born with CLP,^6^, ^7^, ^8^, ^9^, ^10^ the findings are not robust. Few studies have been able to undertake analyses of both exposure and outcome in samples with sufficient power.^16^ In 2015, we used data from the Cleft Care UK study (CCUK) to report on parental perceptions of the child's self‐confidence at 5 years of age^17^ in a centralized cleft care service. The CCUK study collected data on a variety of exposures and outcomes in 268 children born with unilateral CLP. Eight per cent of parents perceived that the child's cleft had adversely affected the child's self‐confidence compared to 19% in the 1998 CSAG study^18^ suggesting a potentially beneficial effect of centralization.

The aims of this study are to extend analysis of the CCUK data to describe psychosocial and behavioural outcomes in more detail, explore variation in outcomes as a function of treatment centre and investigate individual predictors of these outcomes.

## 2 PARTICIPANTS AND METHODS

### 2.1 Study sample

We used data from the CCUK study. This is a UK‐wide cross‐sectional study of 5‐year‐old children born between April 2005 and March 2007 with UCLP. A full description of recruitment procedures and eligibility criteria can be found elsewhere.^17^, ^19^ Briefly, of 359 eligible children, consent for participation was obtained from 268 (75%) children and parents. Ethical approval was obtained (REC reference number: 10/H0107/33, South West 5 REC). Eligible families were invited to attend a designated study clinic. Consent from parents to take part in the study and assent from the children themselves were sought on arrival at the clinic.

### 2.2 Behavioural and psychosocial measures

Two questionnaires were used to collect behavioural and psychosocial data, one concerned with the psychosocial assessment of the child (including the Strengths and Difficulties Questionnaire [SDQ]^20^) and the other with the health and lifestyle (HLQ) of the family. The SDQ was administered by a psychologist when the child and his/her family attended the cleft clinic and parents were asked to complete items about the child's self‐confidence in the HLQ either while they were at the clinic or when they returned home.

### 2.3 Self‐confidence

The child's self‐confidence was based on parental response to the question “Do you feel your child's self‐confidence has been affected by the cleft?” Responses were scored from 1 to 10 where 1 represents a negative effect of the cleft on self‐confidence, 5 represents no difference and 10 represents a positive effect. Scores were grouped so that ratings from 0 to 4 were categorized as a negative effect of the cleft and compared to the remaining reference category (ratings≥5).

### 2.4 Strengths and difficulties

The SDQ comprises 25 parent‐completed items relating to five different domains: emotional (anxiety and depression), conduct, hyperactive and peer‐related behaviour problems as well as prosocial behaviour. Parents were asked to respond to each item using a three‐point response scale from 0 (not true) to 2 (certainly true). Each domain is assessed by five items giving an overall domain scale ranging between 0 and 10. Anchor points for each item in each domain except the prosocial were reversed as appropriate so that high scores represent higher levels of behavioural difficulties. High scores for the prosocial domain represent fewer behavioural difficulties. Individual SDQ domains can also be aggregated to produce an internalizing score (anxiety and depression scores combined), an externalizing score (hyperactive and conduct scores combined) and a total difficulty score which combines all domains except the prosocial domain.

### 2.5 Facial appearance

Full details of the methodology have been reported previously elsewhere.^21^ Briefly, facial appearance was assessed from photographs using a standardized and validated aesthetic outcome assessment tool for the evaluation of cleft lip and palate surgical repairs. An orthodontist rated each cropped image using a five‐point Likert‐type scale (1=Excellent, 2=Good, 3=Fair, 4=Poor or 5=Very Poor). This five‐point ordinal scale was adapted and developed by the Birmingham Institute of Paediatric Plastic Surgery from an existing method.^22^

### 2.6 Speech measures

The data collection methods for speech have been described previously.^23^ Two independent listeners undertook perceptual analysis using the CAPS‐A tool to give a measure of speech intelligibility/distinctiveness, and it is this measure that is used within the analyses of this study. The CAPS‐A also gives a structural score (derived from measures of hypernasality, audible nasal emission, nasal turbulence and the passive category), an articulation measure (derived from the anterior, posterior and non‐oral categories) and a summary score of combining both structure and articulation function. (Findings for the structural and articulation scores are reported online.) Further details of the derivation of these scores are given in Sell et al. (within this supplement).^3^

### 2.7 Statistical analysis—centre‐level variation

Centre‐level variation in self‐confidence and SDQ scores at age 5 was examined using hierarchical regression. Based on these models, we estimated the variance partition coefficient (VPC)—a measure of the proportion of total variation in outcomes that can be attributed to centre and used estimates from the model to predict the mean outcomes in each centre. Likelihood ratio tests were performed to assess whether any observed variation between centres could be attributed to chance. All results are adjusted for differences in age and gender. Full details of the method for examining centre‐level variation are described elsewhere in this supplement.^24^

### 2.8 Statistical analysis—distribution of strengths and difficulty scores

We have reported mean scores and standard deviations (SD) for SDQ scores by age and gender and have compared these with population averages.^25^ In order to allow comparison with the general population, we have provided descriptive SDQ scores categorized according to diagnostic categories (close to average, slightly high, high, very high compared to average).^25^ These categories were created so that, in a population sample, 80% of the population fall into the “close to average” category, 10% in to the “high” category with 5% falling into each of the “high” and “very high” categories.^26^ However, in this study, we used the actual score for each SDQ domain for the inferential analyses.

### 2.9 Statistical analysis—associates of self‐confidence and strengths and difficulty scores

Associations between parent‐reported self‐confidence, SDQ scores and the child's appearance and speech were investigated with cumulative logit regression models, adjusted for gender, age and social deprivation. They were reported using odds ratios, 95% confidence intervals and Wald test *P* values. Facial appearance and speech were dichotomized because some of the descriptive categories had relatively few children in them. Children were therefore categorized as having either a “good appearance” (excellent or good facial appearance) or “poor appearance” (fair, poor or very poor facial appearance) and “good” (excellent or good) or “poor” intelligibility.

## 3 RESULTS

### 3.1 Sample description

Data on self‐confidence and strengths and difficulties were available on 243 and 205 children, respectively. Average SDQ scores and standard deviations (SD) are shown in Table 1 together with population scores for children aged 5‐10 years relevant to each domain^25^ to allow comparison. There is little evidence of gender differences for any of the SDQ domains apart from those for hyperactive behaviour.

**Table 1:** SDQ behaviour scores by gender

SDQ Scores	Mean (SD)		*P*‐value	Mean (SD)		*P*‐value
	Male	Population Average^a^		Female	Population Average^a^	
Hyperactive difficulties score	4.5 (2.7)	4.1 (2.8)	0.03	3.8 (2.6)	3.1 (2.5)	0.008
Emotional difficulties score	1.8 (2)	1.8 (2)	0.50	2.2 (2)	2 (1.9)	0.18
Conduct difficulties score	1.8 (1.7)	1.8 (1.8)	0.50	1.6 (1.7)	1.5 (1.5)	0.29
Poor peer relationships	1.3 (1.6)	1.5 (1.7)	0.05	1.1 (1.5)	1.3 (1.6)	0.11
Prosocial behaviour	8.2 (1.9)	8.4 (1.7)	0.09	8.8 (1.4)	8.9 (1.4)	0.26
Overall externalizing behaviour score	6.3 (3.9)			5.4 (3.9)		
Overall internalizing behaviour score	3 (3)			3.3 (2.9)		
Total behavioural difficulties score	9.3 (5.7)			8.7 (5.5)		

a Population average (aged 5‐10 years old), see Meltzer et al.^25^ (N=10 298 parent reports).

### 3.2 Comparison of strengths of difficulties with the general population

Table 1 shows there is moderate‐to‐strong evidence that mean scores for hyperactive behaviour problems are higher for children born with UCLP than for children in the general population (*P*=.03 and .008 for boys and girls, respectively). Boys born with CLP had higher overall externalizing difficulties scores than girls. In Table 2, we have reported the proportion of children in each domain according to diagnostic category.^25^ In each instance, according to parental ratings of behaviour, at least 62.4% of children born with UCLP had behaviour scores that are close to average for the general population compared to 80% of the general population of children who are categorized as having scores close to average.^25^ Between 15.2% and 17.5% of boys were reported by their parents as having high or very high levels of problems in each behavioural domain and 13.8% and 17.1% of girls were rated as having high or very high levels of both emotional and conduct behaviour problems, respectively. Very high levels of peer problems were also reported for 7.7% of girls.

**Table 2:** Proportion of children in each diagnostic category by gender

Categories^a^	Hyperactivity		Emotion		Conduct		Peer		Prosocial		Total difficulties	
	N (%)		N (%)		N (%)		N (%)		N (%)		N (%)	
	Male	Female	Male	Female	Male	Female	Male	Female	Male	Female	Male	Female
Close to Average	78 (62.4)	42 (70)	63 (72.4)	42 (72.4)	63 (63)	28 (59.5)	48 (64)	28 (71.8)	96 (70.1)	59 (81.9)	98 (77.2)	57 (87.7)
Slightly high	28 (22.4)	13 (21.7)	9 (10.3)	8 (13.8)	9 (9)	11 (23.4)	14 (18.7)	8 (20.5)	17 (12.4)	7 (9.7)	16 (12.6)	2 (3.1)
High	9 (7.2)	2 (3.3)	11 (12.6)	6 (10.3)	11 (11)	6 (12.8)	9 (12)	0 (0)	14 (10.2)	3 (4.2)	6 (4.7)	3 (4.6)
Very high	10 (8)	3 (5)	4 (4.7)	2 (3.5)	4 (4)	2 (4.3)	4 (5.3)	3 (7.7)	10 (7.3)	3 (4.2)	7 (5.5)	3 (4.6)
Total	125 (100)	60 (100)	87 (100)	58 (100)	87 (100)	47 (100)	75 (100)	39 (100)	141 (100)	74 (100)	127 (100)	65 (100)

a Categories for number of problems listed for each SDQ domain. This fourfold classification is created from data for a UK community sample {Meltzer, 2000}. Categories were created so that 80% of the population fall into the “close to average” category, 10% in to the “high” category with 5% falling into each of the “high” and “very high” categories {http://www.sdqinfo.org/py/sdqinfo/b3.py?language=Englishqz(UK)}.

### 3.3 Centre‐level variation

**Figure 1:**
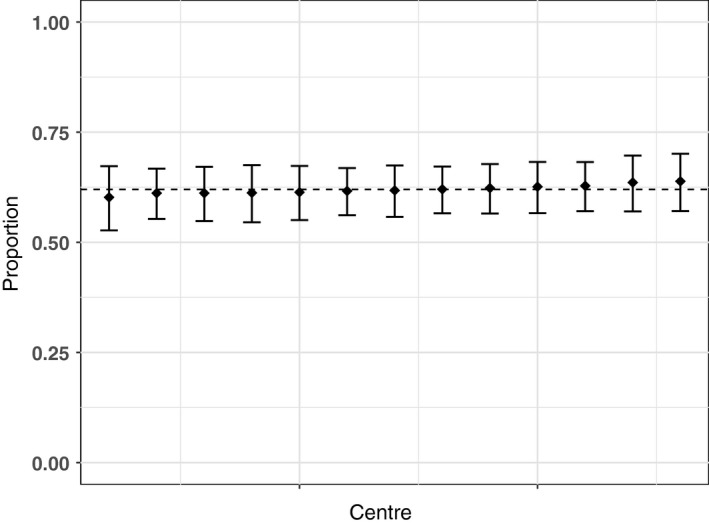
Predicted proportion of children with average hyperactivity in each centre. The bars are 95% confidence intervals, and the dashed line is the predicted mean for the average centre. All results are adjusted for age and sex

**Figure 2:**
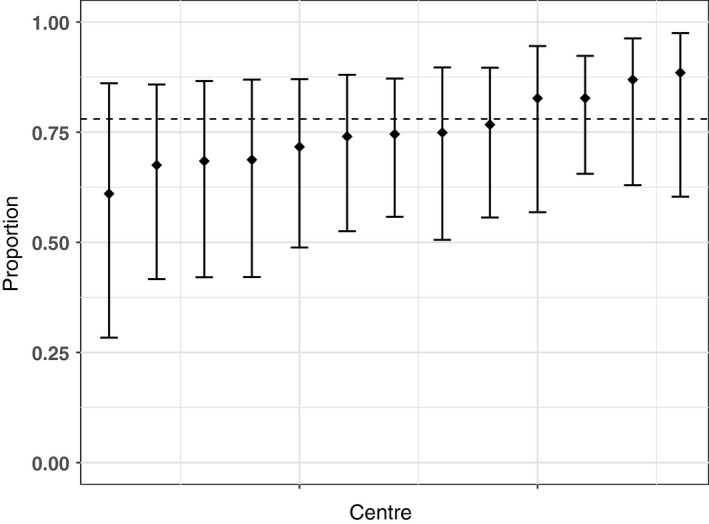
Predicted proportion of children with average Emotions in each centre. The bars are 95% confidence intervals, and the dashed line is the predicted mean for the average centre. All results are adjusted for age and sex

**Figure 3:**
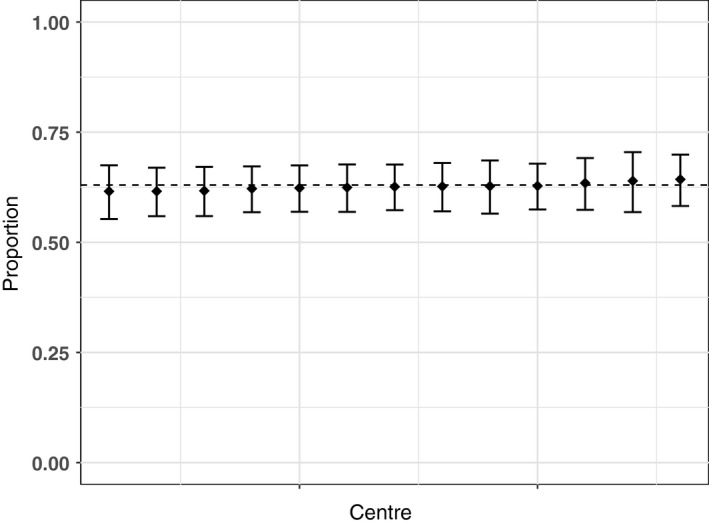
Predicted proportion of children with average conduct behaviour in each centre. The bars are 95% confidence intervals, and the dashed line is the predicted mean for the average centre. All results are adjusted for age and sex

**Figure 4:**
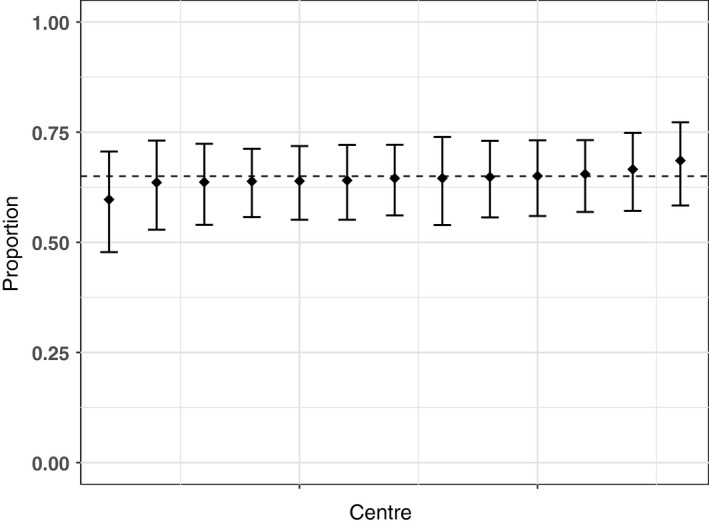
Predicted proportion of children with average peer relationships in each centre. The bars are 95% confidence intervals, and the dashed line is the predicted mean for the average centre. All results are adjusted for age and sex

**Figure 5:**
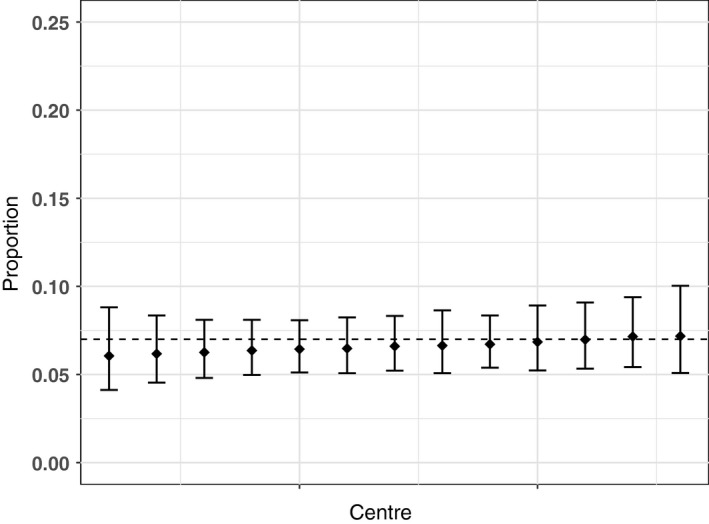
Predicted proportion of children with very low prosocial problems in each centre. The bars are 95% confidence intervals, and the dashed line is the predicted mean for the average centre. All results are adjusted for age and sex

**Figure 6:**
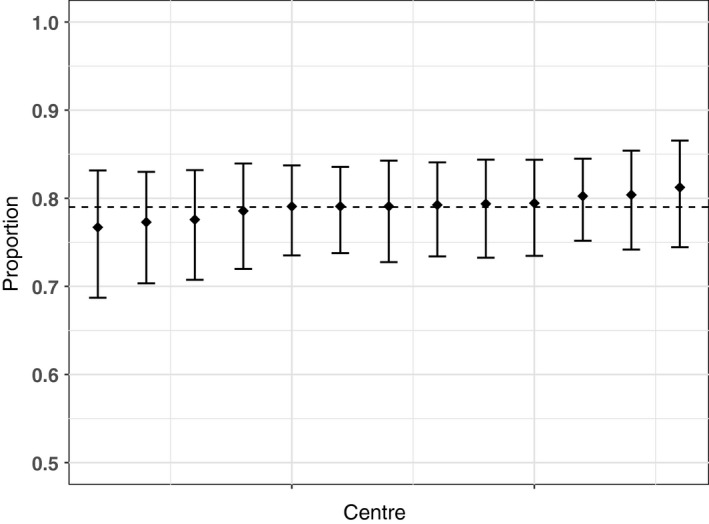
Predicted proportion of children with average Goodman score in each centre. The bars are 95% confidence intervals, and the dashed line is the predicted mean for the average centre

Results of the centre variation analysis are shown in Table 3 and Figures 1, 2, 3, 4, 5, 6. There was no variation in either self‐confidence or SDQ scores. The predicted proportion of children with unaffected self‐confidence based on an “average” centre is 94%. The variance partition coefficient (VPC)—a measure of the proportion of total variation that can be attributed to centre—was reported as 1%. Given that there was no evidence of centre‐level variation, we did not adjust for centre in any of the other analyses in this study.

**Table 3:** Predicted mean with each outcome for the so‐called “average” centre and the between‐centre variability (Variance Partition Coefficient ‐ VPC)

Factor	n	Proportion (95% CI)	VPC	*P*‐value^a^
Self‐confidence				
Unaffected (units)	243	0.94 (0.81, 0.98)	0.011	.90
Strengths & Difficulties				
Hyperactivity (Average)	185	0.62 (0.53, 0.70)	0.02	.90
Emotion (Average)	145	0.75 (0.59, 0.86)	0.015	.90
Conduct (Average)	147	0.63 (0.52, 0.72)	0.02	.90
Peer (Average)	114	0.65 (0.53, 0.76)	0.03	.90
Prosocial (Very low)	209	0.07 (0.04, 0.11)	0.02	.90
Total difficulties (Average)	203	0.80 (0.72, 0.86)	0.03	.90

a The *P*‐value is a test of the null hypothesis that there is no between‐centre variation. All results are adjusted for age and sex.

### 3.4 Associates of self‐confidence and strengths and difficulties scores

Tables 4 and 5, respectively, show the results of the regression analysis to identify associations between child clinical outcomes (facial attractiveness and speech) and self‐confidence and behavioural difficulties as measured by the SDQ. There were no associations between the child's appearance or intelligibility and either their self‐confidence or their behaviour.

**Table 4:** Odds ratios and 95%CI for each domain of behavioural problems as a function of the child's clinical outcomes (facial appearance and speech)—adjusted for age, gender and deprivation score

	Hyperactivity		Emotion	
	Odds (95% CI)	*P*‐value	Odds (95% CI)	*P*‐value
Good Intelligibility	0.84 (0.39, 1.82)	.663	1.55 (0.55, 4.34)	.407
Good Appearance	0.70 (0.34, 1.48)	.353	1.13 (0.46, 2.76)	.787

	Conduct		Peer	
Good Intelligibility	1.15 (0.46, 2.85)	.766	1.36 (0.53, 3.48)	.528
Good Appearance	1.05 (0.47, 2.34)	.903	1.67 (0.68, 4.07)	.262

	Prosocial		Total difficulties	
Good Intelligibility	0.69 (0.31, 1.54)	.363	1.46 (0.56, 3.80)	.433
Good Appearance	0.64 (0.32, 1.27)	.201	0.96 (0.40, 2.28)	.925

**Table 5:** Odds ratios and 95% CI self‐confidence as a function of the child's clinical outcomes (facial appearance and speech)—adjusted for age, gender and deprivation score

	Self‐confidence (not affected)	
	Odds (95% CI)	*P*‐value
Good Intelligibility	1.03 (0.33, 3.63)	.955
Good Appearance	0.37 (0.08, 1.25)	.142

## 4 DISCUSSION

In our study, 5‐year‐old children with UCLP have higher levels of parentally reported behavioural difficulties than the general population as measured by the SDQ. We found no evidence of centre‐level variation in either behavioural problems or self‐confidence within the UK centralized multidisciplinary service. There is also no evidence of any association between child appearance, self‐confidence and behaviour or the intelligibility of their speech, self‐confidence and behaviour.

### 4.1 Consistency with other studies

Our results are consistent with other research reporting higher levels of behavioural difficulties in children born with CLP.^1^, ^5^, ^7^, ^8^, ^9^ Some authors^27^, ^28^ have shown that children with poor language skills are less able to self‐regulate and more likely to display behavioural difficulties, but our findings do not support this conclusion. However, it is possible that other child, parenting or family factors may be associated with reported behavioural difficulties and in part explain the associations we and others have observed.^29^, ^30^, ^31^, ^32^

### 4.2 Strengths and limitations

This study was large (for examination of cleft lip and palate) and nationwide with a good response rate, limited age range, a series of validated key outcomes measured with enough precision to demonstrate improvements over time. However, our work does have a number of limitations. First, the data are cross‐sectional meaning that caution is required when making assumptions about causality. Second, we only recruited families with a child born with UCLP to the study meaning that we cannot generalize to other phenotypic subgroups. Third, although the majority of eligible families consented to participate in the study, there is a relatively large amount of missing data. This is especially true for those items comprising the self‐report questionnaire to be completed at home. Fourth, this study has limited power to detect modest centre‐level variation in treatment and outcome and other effects. Finally, there is the possibility that observed associations reflect confounding that we have not accounted for—for example, as noted above, the parent‐child relationship is likely to influence both speech and child behaviour.

### 4.3 Implications for policy and practice

Our findings have implications for policy and practice. More children in this cohort have higher than average levels of poor behaviour than are found in the general population. Other papers in this supplement^3^, ^33^ highlight the fact that a substantial proportion of five‐year‐olds born with UCLP have hearing and speech problems: in conjunction with the poor behaviour reported here, these children may be unnecessarily disadvantaged. Speaking and hearing skills will impact not only on the family but also on achievement and relationships outside the home: at school and with peers. As noted elsewhere,^27^, ^28^ children with poor speech outcomes are at higher risk of poor behaviour and so it is important to continue the integration of cleft care services so that psychologists, audiologists and speech therapists can work together to manage skills and behaviour and facilitate optimal outcomes for these children. A survey of the cleft service in the UK at the time the CCUK study was undertaken reported variations in cleft care provision regarding the presence of these specialties in cleft teams: while 14/15 teams had a speech and language therapist (SLT) and 14/15 had an ENT surgeon and/or an audiology physician, only 11/14 had access to psychological services. There was also variation in attendance at multidisciplinary team meetings (MDTs): all SLTs, 9/11 psychologists and 7/14 ENT surgeons/audiologists attended MDTs all or most of the time.^34^ Our data suggest a need for psychological support—if teams cannot provide such support where required some, children may be disadvantaged socially and academically as a result.

### 4.4 Research implications

To our knowledge, this is the first study of this size to report on these associations and these findings require replication. If our findings are confirmed, and given the potentially lifelong adverse outcomes associated with behavioural difficulties in childhood (for example reduced social, academic and activity competencies^1^, ^5^), larger longitudinal studies with better measures of potential confounding factors will be required. It may be that the age of children in this study (around 5 years of age) is suboptimal for collecting reliable parental reports of child behaviour given that this is a transitional stage where children start school and are likely to be facing changes in environment, peers and expectations—behaviour might be expected to vary while children learn to adapt. It will also be important to examine the impact of parenting attitudes and behaviour as well as the parent‐child relationship on child psychosocial outcomes. We may be able to address some of these issues using the family, parent and child items included in the health and lifestyle questionnaire that CCUK study parents were asked to complete.

## 5 CONCLUSIONS

Our study showed that children born with UCLP have higher levels of behaviour problems compared to those in the general population. There is no centre variation in these behavioural outcomes and no associations between facial appearance, intelligibility and self‐confidence or behaviour. Further studies are required to replicate and extend these analyses. Given the relatively high levels of behavioural problems in this cohort, children with CLP should have access to psychological support within the current multidisciplinary centralized service, and this support should be provided in conjunction with appropriate speech and language and audiology support.
